# Clinical Features and Outcomes Associated with Angioedema in the Emergency Department

**DOI:** 10.5811/westjem.2019.6.42852

**Published:** 2019-08-06

**Authors:** Benjamin J. Sandefur, Lucas Oliveira J. e Silva, Christine M. Lohse, Kiran A. Goyal, David W. Barbara, Ana Castaneda-Guarderas, Xiao-wei Liu, Ronna L. Campbell

**Affiliations:** * Mayo Clinic, Department of Emergency Medicine, Rochester, Minnesota; † Federal University of Rio Grande do Sul, Department of Medicine, Rio Grande do Sul; ‡ Mayo Clinic, Division of Biomedical Statistics and Informatics, Rochester, Minnesota; § Mayo Clinic, Department of Anesthesiology & Perioperative Medicine, Rochester, Minnesota; ¶ The First Affiliated Hospital of China Medical University, Department of Emergency Medicine, Liaoning, Shenyang

## Abstract

**Introduction:**

Angioedema represents self-limited, localized swelling of submucosal or subcutaneous tissues. While the underlying etiology may be undeterminable in the emergent setting, nonhistaminergic and histaminergic angioedema respond differently to therapeutic interventions, with implications for empiric treatment. Clinical features and outcome differences among nonhistaminergic vs histaminergic angioedema patients in the emergency department (ED) are poorly characterized. We aim to describe the clinical characteristics and outcomes among ED patients with angioedema by suspected etiology.

**Methods:**

This was a 10-year retrospective study of adult ED patients with angioedema, using data abstracted from the electronic health record. We evaluated univariable associations of select clinical features with etiology and used them to develop a multivariable logistic regression model for nonhistaminergic vs histaminergic angioedema.

**Results:**

Among 450 adult angioedema patients, the mean +/− standard deviation age was 57 +/− 18 years, and 264 (59%) were female. Among patients, 30% had suspected nonhistaminergic angioedema, 30% had suspected histaminergic angioedema, and 40% were of unknown etiology. As compared to histaminergic angioedema, nonhistaminergic angioedema was associated with angiotensin-converting enzyme inhibitors (ACEI) or use of angiotensin II receptor blockers (ARB) (odds ratio [OR] [60.9]; 95% confidence interval [CI], 23.16–160.14) and time of onset one hour or more prior to ED arrival (OR [5.91]; 95% CI,1.87–18.70) and was inversely associated with urticaria (OR [0.05]; 95% CI, 0.02–0.15), dyspnea (OR [0.23]; 95% CI, 0.08–0.67), and periorbital or lip edema (OR [0.25]; 95% CI, 0.08–0.79 and OR [0.32]; 95% CI, 0.13–0.79, respectively).

**Conclusion:**

As compared to histaminergic angioedema, patients with nonhistaminergic angioedema were more likely to present one hour or more after symptom onset and take ACEI or ARB medications, and were less likely to have urticaria, dyspnea, or periorbital or lip angioedema. Identification of characteristics associated with the etiology of angioedema may assist providers in more rapidly initiating targeted therapies.

## INTRODUCTION

Angioedema is a self-limited and localized swelling of the submucosal or subcutaneous tissues. This process is caused by a temporary increase in vascular permeability allowing passage of fluid from the intravascular space to the interstitial space, and is mediated through the actions of vasoactive substances, primarily histamine or bradykinin.^1^ Angioedema is non-pitting and is not gravity dependent, and can involve myriad physical locations, including anatomic structures of the upper airway.^2^,^3^ While rare, death due to asphyxiation has been described, and concern about airway compromise is frequently the primary determinant of the initial management and disposition of patients presenting with angioedema to the emergency department (ED).^4^–^7^ Among patients presenting to the ED with angioedema, approximately one-third are associated with angiotensin-converting enzyme inhibitors (ACEI), representing the most common bradykinin-mediated angioedema syndrome encountered in this setting.^8^,^9^ The remainder are comprised of histamine-mediated syndromes and, to a far lesser extent, hereditary and acquired angioedema syndromes related to complement aberrations.^3^,^8^–^12^ Angioedema of unknown etiology has represented a large percentage (30–59%) of cases in previously reported cohorts.^3^,^13^–^15^

Histaminergic angioedema arises from mast cell degranulation and is effectively treated with epinephrine, antihistamines, and corticosteroids.^16^ Epinephrine is the first-line therapy for life-threatening histaminergic and undifferentiated angioedema.^16^ The etiologies of bradykinin-mediated angioedema include hereditary angioedema syndromes (HAE), acquired catabolism of C1 inhibitor (C1-INH), and ACEI-associated angioedema.^16^–^19^ Targeted interventions for acute presentations of hereditary or acquired angioedema due to C1-INH deficiency include C1-INH concentrates (Berinert P; CSL Behring, Marburg, Germany), bradykinin-receptor antagonists (icatibant; Jerini, Berlin, Germany), or plasma kallikrein inhibitors (ecallantide; Dyax Corp, Cambridge, Massachusetts).^1^,^20^ The care of ACEI-associated angioedema in the emergent setting includes acute airway management and discontinuation of the offending medication.^7^,^16^ While initial results among patients receiving icatibant for ACEI-associated angioedema were promising, larger trials evaluating targeted HAE therapies for ACEI-associated angioedema have yielded disappointing results.^17^,^21^–^23^

Laboratory studies are of limited utility in the emergent setting. A normal C4 level in the acute setting reasonably excludes HAE type I and type II, and an elevated tryptase level supports a histaminergic etiology.^1^ However, these studies may not be available in some settings, and even if they were, results would not be available in a timeframe sufficient to guide ED care.^16^,^24^ However, the suspected etiology of angioedema does have pragmatic implications in the ED, where critical decisions regarding empiric therapy, airway management, and patient disposition must be rapidly made.

**Population Health Research Capsule**

What do we already know about this issue?

*Angioedema arises from histamine or bradykinin effect, and the underlying etiology determines response to therapeutic interventions*.

What was the research question?

*Are there clinical features that differentiate ED patients with histaminergic versus nonhistaminergic angioedema*?

What was the major finding of the study?

*Patients with nonhistaminergic angioedema are less likely to present with urticaria, dyspnea, or periorbital or lip edema*.

How does this improve population health?

*Identification of clinical characteristics associated with histaminergic or nonhistaminergic angioedema syndromes may guide emergency providers in initiating treatment*.

Given the differences in pathophysiology and response to targeted therapies of the various angioedema clinical syndromes and the absence of timely laboratory studies that can help differentiate the underlying etiology, an understanding of the differences in clinical features among histaminergic vs nonhistaminergic angioedema may assist the emergency provider in determining the underlying etiology. However, differences in clinical features among histaminergic vs nonhistaminergic angioedema syndromes are not well described. We aimed to describe the clinical features, management, and outcomes of a 10-year cohort of patients who presented with angioedema to a large quaternary ED, identifying clinical factors and outcomes associated with angioedema etiology.

## METHODS

### Study Design, Setting and Participants

Our retrospective cohort study was approved by the Mayo Clinic Institutional Review Board. All adult patients (age ≥ 18 years) evaluated for angioedema in the ED of Mayo Clinic Hospital (Rochester, Minnesota) from January 1, 2005, to December 31, 2014, were eligible for inclusion. The number of cases during the study period determined the study size. The ED at our quaternary care academic institution had an average annual census of 74,000 during the study period. Patients were identified by diagnostic codes for angioedema (*International Classification of Diseases, Ninth Revision [*ICD-9*]* code 995.1), hereditary angioedema (*ICD-9* 277.6), edema of the pharynx or nasopharynx (ICD-9 478.25), or edema of the larynx (ICD-9 478.6). ICD-9 diagnostic codes for anaphylaxis were not used to identify patients; however, patients identified with angioedema and associated anaphylaxis were included. We obtained and reviewed charts of patients with angioedema identified within three days from an ED evaluation. Patients with subjective angioedema (ie, no documented swelling) and angioedema that had resolved prior to ED arrival were excluded. We also excluded patients with swelling caused by another identifiable etiology, such as lymphedema, localized infection, trauma, or inflammatory response from irritant substance. All patients evaluated at our institution were asked for permission to use their medical records for research; those who declined were excluded. Our study adheres to the STROBE (Strengthening the Reporting of Observational studies in Epidemiology) guidelines for reporting observational studies.^25^

### Data Sources and Measurement

We abstracted data from the electronic health record (EHR) using a standardized chart review process.^26^ All ED visits were independently extracted in duplicate by an undergraduate student (K.A.G.) and a medical student (L.O.J.S.). Students were trained by the principal investigator (PI) (B.J.S) on 20 random charts, and coding rules were developed. Investigators met biweekly to discuss inconsistencies or ambiguities with the PI, and these charts were again reviewed in detail to ensure accuracy of coding. We developed additional abstraction instructions as needed to ensure consistent and accurate data. A sample of 75 visits (10.0%) was independently extracted by the PI, and inter-rater reliability with the final data extracted by the students was calculated for key variables using the Cohen’s kappa statistic. Key variables included the following: time of onset; urticaria; airway intervention; disposition; etiology of angioedema in ED; etiology of angioedema at allergy-immunology consultation; and 30-day mortality. Interobserver agreement (kappa) was strong for most variables, and ranged from 0.70 to 1.0.

We collected and managed study data using REDCap (Research Electronic Data Capture, Nashville, TN) electronic data capture tools hosted at Mayo Clinic.^27^

### Variables and outcomes

We defined angioedema as localized subcutaneous or submucosal swelling objectively described by the provider documentation in the EHR. Swelling was required to be present on physical examination documentation. When notes were ambiguous among providers, the documentation of the attending physician was given preference. We classified angioedema into three categories: nonhistaminergic angioedema, histaminergic angioedema, and angioedema of unknown etiology. This classification was determined based on documentation of the suspected cause of angioedema reported by the ED provider, dismissing hospital physician, or allergist-immunologist documentation, when available. The final suspected etiology was based on the allergist-immunologist documentation and diagnosis if the patient had allergist-immunologist evaluation, the hospital dismissal diagnosis if the patient was admitted to the hospital, or the ED provider diagnosis if the patient was not admitted and did not have allergist-immunologist evaluation.

Nonhistaminergic angioedema included ACEI-associated angioedema, HAE type I and type II, acquired angioedema with C1-INH deficiency, and HAE with normal C1-INH. Histaminergic angioedema included patients presenting with angioedema and a temporally-related exposure to a likely allergen (ie, medications, foods, and stinging insects) with rapid development of symptoms, and angioedema with multisystem involvement and documented anaphylaxis. Angioedema was categorized as unknown etiology when clear provider documentation of unknown etiology existed, when no clear etiologic agent could be identified on review of the documentation, and when features or inciting causes of both histaminergic and nonhistaminergic syndromes were documented by the provider and were unable to be reconciled.

We reviewed pertinent documentation from the ED evaluation, prehospital and referring hospital, when applicable, and hospital course, as were any allergy-immunology consultation records. We collected 1) baseline characteristics: demographic information, medical history, medications and allergies; 2) history and physical exam: suspected triggers of angioedema, time of onset relative to ED presentation, location of angioedema, clinical signs and symptoms associated with angioedema; 3) suspected cause of angioedema by the emergency provider; 4) treatment provided in the ED; 5) airway management; 6) ED disposition; 7) suspected cause of angioedema at hospital discharge; 8) hospital length of stay, in-hospital mortality and 30-day mortality; 9) allergy-immunology evaluation (either during an associated hospitalization or within 30 days of the index ED visit) and suspected cause of the angioedema by an allergist-immunologist, when available. We categorized the palate, uvula, and tonsillar pillars as pharyngeal structures and the epiglottis, arytenoids, aryepiglottic folds, false vocal cords and true vocal cords as laryngeal structures.

We defined treatment as any medication or blood product used to treat angioedema, including H1 and H2 antihistamine medications, epinephrine, corticosteroids, albuterol, fresh frozen plasma, and targeted therapies such as C1-INH concentrates, bradykinin-receptor antagonists, or kallikrein inhibitors. The need for tracheal intubation was defined as a tracheal intubation attempt. Fiberoptic laryngoscopies with a bronchoscope prepared for intubation were not categorized as a tracheal intubation attempt unless an attempt to intubate the trachea was documented. ED disposition included home, ED observation, hospital admission (including hospital observation admission), intensive care unit (ICU) admission, and death in the ED. Disposition following ED observation status was collected. In-hospital and 30-day mortality included deaths for all causes.

### Statistical Methods

We summarized continuous variables with means and standard deviations (SD). Categorical features were summarized with frequency counts and percentages. Comparisons of features by etiology were evaluated using analysis of variance, Kruskal-Wallis, chi-square, and Fisher’s exact tests. We further evaluated associations of select features with type of etiology (histaminergic vs nonhistaminergic) using logistic regression models and summarized them with odds ratios (OR) and 95% confidence intervals (CI). Age was analyzed as a continuous variable. The OR represents the odds of nonhistaminergic angioedema for each 10-year increase in age (Table 3). Multivariable models were developed using forward selection. We performed statistical analyses using SAS version 9.4 (SAS Institute; Cary, NC). All tests were two-sided, and p-values <0.05 were considered statistically significant.

## RESULTS

### Participants

We identified 752 ED visits potentially eligible for our study, of which 450 visits among 400 distinct patients met our inclusion criteria and were available for analysis. We excluded visits with presentations attributable to an infectious etiology (32); isolated urticaria (25); complications of a malignancy or mass (23); a traumatic, burn-related, or caustic etiology (13); subjective angioedema (13); post-procedural swelling (6); anaphylaxis without angioedema (3); lymphedema (1); or internal jugular vein thrombosis (1). We excluded visits if the patient left prior to evaluation (4) or had angioedema in the prehospital setting that had resolved upon ED evaluation (15). Three patients who declined research authorization were excluded. The remaining 163 excluded ED visits were unrelated to angioedema, and were captured in the ICD-9 diagnostic code query due to a prior angioedema diagnosis or subsequent development of angioedema during the hospitalization or a future encounter.

### Descriptive Data

The annual rate of angioedema was 0.6 per 1000 ED visits. The mean +/− SD age at presentation was 57 +/− 18 years, and 264 (59%) were female (Table 1). A majority of our cohort was white (89%). African Americans represented 6% of our cohort, and African Americans comprised 4.4% of all patients presenting to our ED during the study period. Eighty-seven (19%) patients were transported by ambulance. Hypertension (61%) was the most common comorbidity, and 45% of patients reported a prior episode of angioedema.

Steroids were the most commonly administered medications (83%) followed by H1 antihistamine medications (79%). Epinephrine was administered in 34% of encounters. Tracheal intubation was required in 33 patients (7%). Patients were frequently discharged to home directly from the ED (38%) or from an ED observation unit (32%). ICU admission occurred in 78 patients (17%). Among the 154 patients who were admitted to an ED observation unit 145 (94%) were discharged, five (3%) were admitted to general care, and four (3%) were admitted to an ICU. A total of 226 (50%) patients had allergy-immunology consultation in the inpatient setting or upon outpatient follow-up. No in-hospital deaths were noted, and mortality within 30 days was rare (1%). No deaths were due to complications of angioedema.

### Outcome Data and Main Results

We compared clinical features and outcomes by etiology of angioedema (nonhistaminergic vs histaminergic vs unknown) among all patients in our cohort (Supplemental Appendix). We identified a probable etiology of angioedema in 60% of patient encounters. We found similar frequencies of nonhistaminergic (30%) and histaminergic (30%) angioedema, and in 40% of patients the etiology of angioedema could not be identified. The specific underlying suspected etiology of the angioedema episodes are summarized in Table 2. ACEI-associated angioedema was the most common cause of nonhistaminergic angioedema. Medication hypersensitivity represented the most common cause of histaminergic angioedema.

Table 3 summarizes univariable associations of clinical features and outcomes among the subset of patients with suspected nonhistaminergic vs histaminergic angioedema among our cohort (n=271). Patients presenting with nonhistaminergic angioedema were more likely to be older than those with histaminergic angioedema, more likely to have had symptoms one hour or more prior to ED arrival, and more likely to have tongue or soft palate swelling. ACEI medication use, hypertension, and diabetes were more common among patients diagnosed with nonhistaminergic angioedema. Periorbital angioedema, lip angioedema, and urticaria were less likely among patients with nonhistaminergic angioedema compared to histaminergic.

Patients with nonhistaminergic angioedema were more likely to be admitted to the ICU (OR [2.58]; 95% CI, 1.35–4.93) compared to a non-ICU disposition (home, ED observation and hospital admission) than those with histaminergic angioedema. Those with upper airway involvement, defined as angioedema of the larynx or tongue, were more likely to require ICU admission (OR [11.27]; 95% CI, 5.87–21.63). ICU admission also was more frequent in patients with nonhistaminergic angioedema (OR [2.18]; 95% CI,1.32–3.61) than a combined subset of histaminergic angioedema and angioedema of unknown etiology, an association that remained after stratification by involvement of the upper airway with angioedema (OR [1.85]; 95% CI, 1.07–3.20).

We developed a multivariable model using a prespecified list of candidate predictor variables (Table 4). ACEI or angiotensin II receptor blockers (ARB) use (OR [60.9]; 95% CI, 23.16–160.14) and presentation one hour or more from symptom onset (OR [5.91]; 95% CI,1.87–18.70) were associated with nonhistaminergic angioedema syndromes. Urticaria (OR [0.05]; 95% CI, 0.02–0.15), dyspnea (OR [0.23]; 95% CI, 0.08–0.67), and periorbital or lip angioedema on physical examination (OR [0.25]; 95% CI, 0.08–0.79 and OR [0.32]; 95% CI, 0.13–0.79, respectively) were inversely associated with nonhistaminergic angioedema.

## DISCUSSION

We describe the clinical features, management, and outcomes of a large, 10-year cohort of adult patients with angioedema presenting to a quaternary-care ED setting. Among 450 ED presentations for angioedema, 30% represented suspected nonhistaminergic angioedema, 30% represented suspected histaminergic angioedema, and in the remaining patients the etiology could not be definitively categorized. Half of the patients in our cohort were evaluated by an allergist-immunologist after ED care. As compared to histaminergic angioedema, nonhistaminergic angioedema was associated with ACEI medication use, earlier symptom onset relative to ED arrival, tongue and soft palate swelling, and ICU admission, and was inversely associated with periorbital angioedema, lip angioedema, dyspnea, and urticaria.

Our patients had a mean age of 57 years and a subtle female predominance, comparable to existing published cohorts of patients with angioedema.^2^,^9^,^14^,^28^,^29^ Nonhistaminergic angioedema represented 30% of our cases, which is consistent with previous reports of ACEI-induced angioedema comprising 30–40% of angioedema cohorts.^8^,^9^,^11^ We were unable to identify an etiology of angioedema in 40% of our population. This finding is comparable to reports of angioedema of unknown etiology representing 30–50% of angioedema patients in similar cohorts.^3^,^13^,^14^ We did not assume a patient to have nonhistaminergic angioedema based upon the use of an ACEI or ARB medication alone. Existing studies have differed in regard to the assignment of ACEI-associated angioedema, whether based upon the presence of ACEI 30,31 or assigned by documented clinician or investigator judgment during chart review.^2^,^5^,^8^,^9^,^14^ We chose to use the judgment and diagnosis assigned by the clinician upon discharge or, when possible, allergist-immunologist at follow-up. Given our approach, ACE inhibitors and ARB medications were taken by some patients with angioedema categorized as histaminergic or unknown etiology. In support of this approach is our observation that approximately 20% of patients in the histaminergic category were using an ACEI or ARB in the overall ED cohort and among the subset of patients who had allergy-immunology consultation.

In a multivariable analysis of the subset patients with suspected nonhistaminergic and histaminergic angioedema, we identified use of an ACEI or ARB medication and the presence of urticaria as the strongest associations with these subgroups, respectively. Time of onset one hour or more from ED presentation was associated with nonhistaminergic angioedema; and dyspnea and angioedema involving the periorbital region or lips were associated with histaminergic syndromes. That urticaria, present in 26% of the overall cohort, is associated with a suspected histaminergic etiology of angioedema is expected; however, we identified patients with suspected nonhistaminergic angioedema and angioedema of unknown etiology who also exhibited urticaria (7% and 26%, respectively). These findings, which could raise concern about the accuracy of classification of angioedema patients, have been noted in similar, published angioedema cohorts.^32^ Felder and colleagues noted approximately 30% of those with angioedema of unknown etiology and 9.1% of patients with angioedema secondary to C1-INH deficiency were noted to have urticaria at presentation, and only slightly lower prevalence among those with ACEI-associated angioedema.^32^ It is also possible that erythema marginatum, sometimes seen in nonhistaminergic angioedema, could be mistaken for urticaria by clinicians.^33^

The rate of tracheal intubation was 7% among all patients presenting to our ED with angioedema. The rate of tracheal intubation among published angioedema cohorts ranges from 5–35%.^2^,^3^,^5^,^8^,^9^,^13^,^30^,^34^,^35^ Our findings are consistent with cohorts of patients presenting with angioedema to an ED setting.^9^,^34^ Studies that have identified patients treated for angioedema in a hospital system by diagnosis-related group code, not exclusive to an ED population, have reported higher rates of tracheal intubation.^2^,^3^,^8^,^13^ This is expected given inclusion of patients directly admitted from other facilities for ICU care. Studies focusing on admitted patients with angioedema have expectedly reported higher intubation rates.^5^,^30^,^35^ Zirkle and Bhattacharyya found an intubation rate of 34.8% among their cohort of admitted patients, modestly higher than an intubation rate of 24.6% among admitted patients in our cohort.^35^

Smith and colleagues, in a study examining the burden of angioedema on EDs in the United States, demonstrated tracheal intubation to be a predictor of angioedema due to antihypertensive medication effect.^29^ McMormick and colleagues reported ACEI use as a significant predictor of airway intervention.^30^ Tracheal intubation rates did not differ by etiology in our cohort.

Patient disposition following ED evaluation differed based upon suspected etiology in our univariable analysis, with nonhistaminergic angioedema patients more frequently requiring ICU care. Ishoo and colleagues found that nearly half of ACEI-associated angioedema patients were admitted to the ICU.^3^ Our lower rate of ICU admission may reflect increased utilization of ED observation units, 32% among our cohort, for this population over the past 15 years.^16^,^36^ Banerji and colleagues reported a rate of observation admission and subsequent discharge of 18% among angioedema patients presenting to academic EDs between 2003 and 2005.^9^ Chiu and colleagues noted a slightly higher admission rate in patients with ACEI-induced angioedema, although this difference was not statistically significant.^2^ An association between nonhistaminergic angioedema and admission to an ICU level of care is logical, given a predilection of ACEI-associated angioedema to involve the upper airway^9^,^10^,^17^,^37^ and the association between upper airway involvement and ICU admission.^5^ In an analysis stratified by upper airway involvement, we found that patients with nonhistaminergic angioedema remained more likely to require ICU admission. This finding may be due to the relatively prolonged duration of ACEI-associated angioedema and its refractory nature to conventional therapies as compared to histaminergic angioedema.

## LIMITATIONS

We conducted our study at a single academic institution; thus, additional research is needed to determine the applicability of our findings to other settings. The retrospective design led to the inherent limitation of obtaining data from an existing medical record. We developed and used a standardized data abstraction tool and created rules related to each data field to minimize inconsistency. We obtained our cohort by searching for ICD-9 codes related to angioedema as have been used in prior studies, and it is possible that this approach may have led to missed cases of angioedema. We categorized patients broadly into histaminergic, nonhistaminergic, or unknown based upon available documentation. Patients in the unknown category lacked compelling evidence at the time of presentation, during hospital admission, or upon follow-up to allow for determination of suspected etiology. Our findings might have been different if we knew with certainty into which group these patients fell; however, the trichotomy we have described approximates the uncertainty experienced in clinical practice and is similar to other reported cohorts.^3^

As our study was observational and retrospective, few patients had C4, tryptase, or C1-INH levels obtained. This is a limitation also present in most existing published ED cohorts. Future, prospective, ED-based studies would benefit from obtaining C4 and tryptase levels at the point of care to better ensure the precision of etiology determination. For example, it is possible that an ACEI might unmask a previously undiagnosed case of HAE or acquired angioedema, although the categorization of nonhistaminergic would remain unchanged. Lastly, our patient population includes a smaller number of African-American patients as compared to previously reported cohorts. Given the 3–4.5 fold increased incidence of ACEI-associated angioedema in African-Americans, our findings may not be generalizable to populations with differing demographics.

## CONCLUSION

In a large cohort of angioedema patients presenting to a quaternary-care ED, similar frequencies of nonhistaminergic angioedema and histaminergic angioedema were noted, and in 40% of patients an etiology could not be established. Among patients with an identified etiology of angioedema, ACEI medication use and urticaria were the strongest predictors of nonhistaminergic and histaminergic angioedema, respectively. As compared to histaminergic angioedema, patients with nonhistaminergic angioedema were more likely to present for care more than one hour from symptom onset, and less likely to present with dyspnea or angioedema of the periorbital region or lips. Identification of these characteristics upon presentation may guide emergency providers in initiating empiric treatment.

## Supplementary Information



## Figures and Tables

**Table 1:** Features of emergency department (ED) patients presenting with angioedema.

Feature	n=450; n (%)
Age at visit (Mean ± SD)	56.8 ± 17.9
Sex	
Female	264 (59)
Race	
White	398 (89)
African-American	25 (6)
All others	25 (6)
Comorbidity (N=449)*	
Angioedema history	200 (45)
COPD	34 (8)
Asthma	49 (11)
Hypertension	272 (61)
Diabetes	105 (23)
Medications	
Neither	255 (57)
ACEI	174 (39)
ARB	19 (4)
ACEI and ARB	2 (<1)
ACEI duration (N=167)	
<1 month	16 (10)
1–6 months	12 (7)
6–12 months	15 (9)
>12 months	124 (74)
Family history of angioedema (N=269)	
Transport by EMS	
Time of onset (N=449)	
In the ED	8 (2)
<1 hour	72 (16)
1–6 hours	245 (55)
6–12 hours	56 (12)
>12 hours	68 (15)
Presenting symptoms*	
Hoarseness	21 (5)
Voice change	76 (17)
Stridor	8 (2)
Drooling	13 (3)
Facial swelling	4 (1)
Periorbital swelling	74 (16)
Lip swelling	261 (58)
Tongue swelling	176 (39)
Shortness of breath	68 (15)
Abdominal pain	5 (1)
Limb swelling	8 (2)
Syncope	3 (1)
Cardiopulmonary arrest	2 (<1)
Urticaria	117 (26)
Wheezing	29 (6)
Objective location of angioedema (N=449)*	
Face	124 (28)
Periorbital	74 (16)
Lips	262 (58)
Uvula	42 (9)
Soft palate	14 (3)
Pharynx	52 (12)
Floor of mouth	1 (<1)
Tongue	177 (39)
Larynx	29 (6)
Neck	8 (2)
Abdomen	5 (1)
Genitalia	1 (<1)
Limbs	33 (7)
Treatment*	
H1 antihistamine	356(79)
H2 antihistamine	230 (51)
Epinephrine	153 (34)
Corticosteroid	372 (83)
Nebulized albuterol	41 (9)
Fresh-frozen plasma	6 (1)
Berinert © (C1 Esterase Inhibitor [Human])	5 (1)
Other‡	4(1)
Intubation	33 (7)
Disposition	
Home	171 (38)
ED observation	145 (32)
Hospital admission	56 (12)
ICU admission	78 (17)
Death in hospital	0
Death within 30 days (N=422)	3 (1)

* Patient can be included in more than one group.

‡ Includes one patient each with blinded study drug, ecallantide, aminocaproic acid, and tranexamic acid, respectively.

*COPD*, chronic obstructive pulmonary disease; *ACEI*, angiotensin-converting enzyme inhibitor; *ARB*, angiotensin II receptor blockers;

*EMS*, emergency medical services; *ED*, emergency department; *ICU*, intensive care unit.

**Table 2:** Summary of final angioedema etiology, N=450.

Final angioedema etiology	n (%)
Nonhistaminergic angioedema	136 (30)
ACEI-associated	118 (26)
ARB-associated	5 (1)
HAE with C1-INH deficiency	8 (2)
HAE with normal C1-INH	3 (<1)
Acquired angioedema with C1-INH deficiency	2 (<1)
Histaminergic angioedema	135 (30)
Medication Allergy	74 (16)
Histaminergic NOS	43 (10)
Food Allergy	16 (4)
Insect Sting	2 (<1)
Unknown	179 (40)

*ACEI*, angiotensin-converting enzyme inhibitor; *HAE*, hereditary angioedema; *C1-INH*, C1 esterase inhibitor; *NOS*, not otherwise specified.

**Table 3:** Univariable associations with final etiology: nonhistaminergic versus histaminergic angioedema.

Feature*	n=271 OR (95% CI)	P-value
Age at visit	1.47 (1.26–1.71)†	<0.001
Sex		
Female	1.0 (reference)	
Male	1.11 (0.69–1.80)	0.66
Race		
White	1.0 (reference)	
African-American	1.51 (0.48–4.75)	0.48
All others	0.29 (0.09–0.92)	0.035
Comorbidity		
Angioedema history	0.98 (0.60–1.61)	0.93
COPD	3.47 (1.23–9.75)	0.019
Asthma	1.14 (0.55–2.39)	0.72
Hypertension	17.57 (8.83–34.95)	<0.001
Diabetes	2.36 (1.34–4.17)	0.003
Medications		
Neither	1.0 (reference)	
ACEI, ARB, or ACEI and ARB	39.67 (19.43–81.0)	<0.001
Transfer from another hospital	2.75 (0.95–7.94)	0.062
Transport by EMS	1.09 (0.59–2.02)	0.78
Time of onset		
In the ED or <1 hour	1.0 (reference)	
≥1 hour	3.95 (1.91–8.16)	<0.001
Presenting symptoms		
Hoarseness	0.65 (0.18–2.36)	0.51
Voice change	1.80 (0.93–3.47)	0.080
Drooling	1.68 (0.39–7.17)	0.48
Shortness of breath	0.64 (0.33–1.23)	0.18
Urticaria	0.08 (0.04–0.18)	<0.001
Wheezing	0.43 (0.16–1.18)	0.10
Objective location of angioedema		
Face	0.92 (0.54–1.56)	0.76
Periorbital	0.27 (0.14–0.53)	<0.001
Lips	0.53 (0.32–0.86)	0.011
Uvula	1.40 (0.54–3.59)	0.49
Soft palate	9.50 (1.19–76.02)	0.034
Pharynx	1.27 (0.57–2.83)	0.56
Tongue	2.50 (1.51–4.14)	<0.001
Larynx	1.34 (0.45–3.98)	0.59
Limbs	0.75 (0.32–1.76)	0.50
Treatment		
H1 antihistamine	0.62 (0.34–1.12)	0.11
H2 antihistamine	0.96 (0.59–1.54)	0.86
Epinephrine	0.77 (0.47–1.26)	0.29
Corticosteroid	0.58 (0.31–1.09)	0.090
Nebulized albuterol	0.69 (0.32–1.47)	0.33
Intubation	2.12 (0.87–5.13)	0.10
Disposition		
Home	1.0 (reference)	
ED observation	0.85 (0.48–1.52)	0.58
Hospital admission	0.70 (0.32–1.53)	0.36
ICU admission	2.28 (1.12–4.66)	0.024
Disposition		
Home/ED observation/hospital admission	1.0 (reference)	
ICU admission	2.58 (1.35–4.93)	0.004

* Only select features of interest present in >5 patients were included in the modeling.

† Odds ratios and 95% confidence intervals represent a 10-unit increase.

*COPD*, chronic obstructive pulmonary disease; *ACEI*, angiotensin-converting enzyme inhibitor; *ARB*, angiotensin II receptor blockers; *EMS*, emergency medical services; *ED*, emergency department; *ICU*, intensive care unit.

**Table 4:** Multivariable associations with final etiology: nonhistaminergic versus histaminergic angioedema.

Feature*	n=271 OR (95% CI)	P-value
Medications		
Neither	1.0 (reference)	
ACEI, ARB, or ACEI and ARB	60.90 (23.16–160.14)	<0.001
Time of onset		
In the ED or <1 hour	1.0 (reference)	
≥1 hour	5.91 (1.87–18.70)	0.003
Presenting symptoms		
Shortness of breath	0.23 (0.08–0.67)	0.007
Urticaria	0.05 (0.02–0.15)	<0.001
Objective location of angioedema		
Periorbital	0.25 (0.08–0.79)	0.018
Lips	0.32 (0.13–0.79)	0.013

* Only select features of interest present in >5 patients were included in the modeling.

*OR*, odds ratio; *CI*, confidence interval; *ACEI*, angiotensin-converting enzyme inhibitor; ARB, angiotensin II receptor blockers; ED, emergency department.
