# Supplementary material for: Clinical Features and Outcomes Associated with Angioedema in the Emergency Department
Source: West J Emerg Med. 2019 Aug 6;20(5):760–9. doi: 10.5811/westjem.2019.6.42852 (PMC6754201; doi:10.5811/westjem.2019.6.42852)
Supplement: Supplementary file 1 [file wjem-20-760-s001.docx]

**Supplemental Appendix.** Comparison of features by final etiology among ED patients presenting with angioedema.

|  | All  N=450 | Nonhistaminergic  N=136 | Histaminergic  N=135 | Unknown Etiology  N=179 |  |
| --- | --- | --- | --- | --- | --- |
|  |  |  |  |  |  |
| Feature | *Mean +/- SD* | | | | P-value^†^ |
| Age at visit | 56.8 +/- 17.9 | 63.5 +/- 15.1 | 52.1 +/- 19.0 | 55.2 +/- 17.5 | <0.001 |
| Sex | *n (%)* | | | |  |
| Female | 264 (59) | 76 (56) | 79 (59) | 109 (61) | 0.67 |
| Race (*N=448*)  White  African-American  All others | 398 (89)  25 (6)  25 (6) | 124 (91)  8 (6)  4 (3) | 117 (87)  5 (4)  13 (10) | 157 (89)  12 (7)  8 (5) | 0.11 |
| Comorbidity (*N=449*)*  Angioedema history  COPD  Asthma  Hypertension  Diabetes | 200 (45)  34 (8)  49 (11)  272 (61)  105 (23) | 49 (36)  16 (12)  17 (13)  124 (91)  46 (34) | 49 (37)  5 (4)  15 (11)  50 (37)  24 (18) | 102 (57)  13 (7)  17 (10)  98 (55)  35 (20) | <0.001  0.042  0.71  <0.001  0.003 |
| Medications  Neither  ACEI  ARB  ACEI and ARB | 255 (57)  174 (39)  19 (4)  2 (<1) | 13 (10)  113 (83)  8 (6)  2 (1) | 109 (81)  21 (16)  5 (4)  0 | 133 (74)  40 (22)  6 (3)  0 | <0.001 |
| ACEI duration (*N=167*)  <1 month  1-6 months  6-12 months  >12 months | 16 (10)  12 (7)  15 (9)  124 (74) | 13 (12)  11 (10)  14 (13)  72 (65) | 1 (5)  0  0  19 (95) | 2 (5)  1 (3)  1 (3)  33 (89) | 0.002 |
| Family history of angioedema (*N=269*) | 14 (5) | 8 (10) | 1 (1) | 5 (4) | 0.049 |
| Transport by EMS | 87 (19) | 26 (19) | 24 (18) | 37 (21) | 0.81 |
| Time of onset (*N=449*)  In the ED  <1 hour  1-6 hours  6-12 hours  >12 hours | 8 (2)  72 (16)  245 (55)  56 (12)  68 (15) | 1 (1)  10 (7)  81 (60)  21 (16)  22 (16) | 5 (4)  30 (22)  68 (50)  13 (10)  19 (14) | 2 (1)  32 (18)  96 (54)  22 (12)  27 (15) | 0.011 |
| Presenting symptoms*  Hoarseness  Voice change  Stridor  Drooling  Facial swelling  Periorbital swelling  Lip swelling  Tongue swelling  Shortness of breath  Abdominal pain  Limb swelling  Syncope  Cardiopulmonary arrest | 21 (5)  76 (17)  8 (2)  13 (3)  4 (1)  74 (16)  261 (58)  176 (39)  68 (15)  5 (1)  8 (2)  3 (1)  2 (<1) | 4 (3)  28 (21)  2 (1)  5 (4)  2 (1)  13 (10)  71 (52)  66 (49)  18 (13)  5 (4)  3 (2)  1 (1)  0 | 6 (4)  17 (13)  3 (2)  3 (2)  1 (1)  38 (28)  90 (67)  36 (27)  26 (19)  0  2 (1)  1 (1)  2 (1) | 11 (6)  31 (17)  3 (2)  5 (3)  1 (1)  23 (13)  100 (56)  74 (41)  24 (13)  0  3 (2)  1 (1)  1 (1) | 0.41  0.21  0.91  0.83  0.83  <0.001  0.041  <0.001  0.27  0.005  1.0  1.0  0.83 |
| Urticaria | 117 (26) | 9 (7) | 62 (46) | 46 (26) | <0.001 |
| Wheezing | 29 (6) | 6 (4) | 13 (10) | 10 (6) | 0.18 |
| Objective location of angioedema (*N=449*)*  Face  Periorbital  Lips  Uvula  Soft palate  Pharynx  Floor of mouth  Tongue  Larynx  Neck  Abdomen  Genitalia  Limbs | 124 (28)  74 (16)  262 (58)  42 (9)  14 (3)  52 (12)  1 (<1)  177 (39)  29 (6)  8 (2)  5 (1)  1 (<1)  33 (7) | 37 (27)  13 (10)  71 (52)  11 (8)  9 (7)  15 (11)  0  66 (49)  8 (6)  3 (2)  5 (4)  0  10 (7) | 39 (29)  38 (28)  91 (67)  8 (6)  1 (1)  12 (9)  0  37 (27)  6 (4)  2 (1)  0  0  13 (10) | 48 (27)  23 (13)  100 (56)  23 (13)  4 (2)  25 (14)  1 (1)  74 (42)  15 (8)  3 (2)  0  1 (1)  10 (6) | 0.92  <0.001  0.030  0.091  0.024  0.36  1.0  0.001  0.35  1.0  0.005  1.0  0.40 |
| Treatment*  H1 antihistamine  H2 antihistamine  Epinephrine  Corticosteroid  Nebulized albuterol  Fresh-frozen plasma  Berinert  Other^‡^ | 356 (79)  230 (51)  153 (34)  372 (83)  41 (9)  6 (1)  5 (1)  4 (1) | 102 (75)  65 (48)  47 (35)  106 (78)  13 (10)  5 (4)  2 (1)  4 (3) | 112 (83)  66 (49)  55 (41)  116 (86)  18 (13)  0  0  0 | 142 (79)  99 (55)  51 (28)  150 (84)  10 (6)  1 (1)  3 (2)  0 | 0.27  0.35  0.075  0.19  0.060  0.025  0.39  0.016 |
| Intubation | 33 (7) | 16 (12) | 8 (6) | 9 (5) | 0.057 |
| Disposition  Home  ED observation  Hospital admission  ICU admission | 171 (38)  145 (32)  56 (12)  78 (17) | 47 (35)  40 (29)  14 (10)  35 (26) | 49 (36)  49 (36)  21 (16)  16 (12) | 75 (42)  56 (31)  21 (12)  27 (15) | 0.055 |
| Disposition  Home/ED observation/hospital admission  ICU admission | 372 (83)  78 (17) | 101 (74)  35 (26) | 119 (88)  16 (12) | 152 (85)  27 (15) | 0.006 |
| Death in hospital | 0 | 0 | 0 | 0 | NA |
| Death within 30 days (*N=422*) | 3 (1) | 0 | 2 (2) | 1 (1) | 0.39 |
|  |  |  |  |  |  |
| *Patient can be included in more than one group.  ^†^P-value for type I vs II vs III.  ^‡^Includes 1 patient each with blinded study drug, ecallantide, aminocaproic acid, and tranexamic acid, respectively.  NA=not applicable | | | | | |

*ED*, emergency department; *EMS*, emergency medical services; *ICU*, intensive care unit; *ACEI*, angiotensin-converting enzyme inhibitor; *ARB*, angiotensin II receptor blockers; *COPD*, chronic obstructive pulmonary disorder.
